# Consensus decision-making in CCAMLR: Achilles’ heel or fundamental to its success?

**DOI:** 10.1007/s10784-021-09561-4

**Published:** 2022-04-07

**Authors:** Lynda Goldsworthy

**Affiliations:** 1 Institute for Marine and Antarctic Studies, University of Tasmania, Hobart, TAS Australia; 2 Centre for Marine Socioecology, Hobart, TAS Australia

**Keywords:** Southern Ocean, CCAMLR, Consensus, Veto, Multilateral organisations, Decision making

## Abstract

The Commission for the Convention on the Conservation of Antarctic Marine Living Resources is the body responsible for the conservation and management of most species in the Southern Ocean. The Convention mandates that decisions be made by consensus agreement of its Members. This approach has been largely successful in delivering strong management decisions across both complex issues and widely ranging national interests. However, recent failures to progress the implementation of a network of marine protected areas or to agree any concrete response actions to climate impacts raise concerns about its effectiveness. This paper reviews the level of uptake of Member-driven proposals and then examines examples of proposals that were not resolved within the usual three years to analyse the processes utilised by Members to find resolution. It concludes that CCAMLR has been successful in reaching agreements when focusing on fisheries management but less so on issues within its broader conservation mandate, such as area protection for biodiversity purposes or non-fishery management focused scientific study, or for issues that are perceiv ed to extend the competency of the Convention. It notes that CCAMLR lacks operational mechanisms to facilitate agreement in the absence of compromise text or when one or two Members cannot accept a proposal.

## Introduction

The Convention on the Conservation of Antarctic Marine Living Resources (CAMLR Convention) was adopted in 1980 by the Consultative Parties to the Antarctic Treaty to manage the expanding interest in harvesting marine resources the Southern Ocean. Its objective—the conservation of Antarctic marine living resources—includes rational uses such as fishing and associated activities if these are conducted according to science-driven precautionary and ecosystem-based principles specified in the Convention.

Article XII of the Convention states that the Commission to the Convention (CCAMLR) shall use consensus to determine all decisions ‘on matters of substance’ (CCAMLR, 1980). This approach was inherited from the Antarctic Treaty, which embraced consensus decision-making to deal with legal and political differences around territorial sovereignty (Scully, 2011).

Many multilateral arrangements use consensus to make decisions, at least for some elements of their work, including the United Nations General Assembly (UNGA), the Antarctic Treaty (AT), the Convention on Biological Diversity (CBD), and several regional fisheries management organisations (RFMOs). This is partly because there are clear benefits from its use, including the perception of a democratic and thus more legitimate process, and the development of collective commitment to the outcome (Ansell & Gash, 2007, 2012; Carter, 2018; Zartman, 1994).

Carter (2018) defines the consensus outcome for multilateral organisations as that which ‘is achieved from a non-voting decision-making process that involves negotiating disagreements of interests, values and ideas among three or more party states collaboratively’ (p. 10). Thus, consensus decision-making as a process relies heavily on active participation, trust that all participants are working towards a common goal (in the case of CCAMLR, delivery of the objective of the Convention) and are committed to the delivery of the agreement once reached (Bressen, 2012; Hefte, 2021). Turner et al. (2008) note that the CAMLR Convention does not provide a specific definition for consensus but conclude that it can be taken to be ‘the absence of a formal objection’ based on the norms practised in the Antarctic Treaty Consultative Meeting and the inclusion of this definition in a related AT instrument.^1^

The process of working towards an agreement also exposes many challenges, including time taken to reach agreement, the level of compromise required and the capacity for just one participant to block progress (Martinez & Montero, 2007). Such challenges are exacerbated when participants don’t share a common goal or purpose.

While CCAMLR struggled in its early years to adopt proposals advocated by many to address recovery of depleted species and control fishing (Vicuña, 1991), by 1990 the Commission had established a more constructive approach. Since then, CCAMLR has agreed many decisions and is widely seen as a leader in the application of a science-driven precautionary approach based on monitoring of indicator species (e.g. Constable, 2011; Kock et al., 2007; Miller, 2013; Miller & Slicer, 2014; Willock & Lack, 2006). According to the CCAMLR website,^2^ major successes include significantly reducing illegal, unreported and unregulated (IUU) fishing activities occurring within the Convention area, initiating the implementation of a network of marine protected areas (MPAs), substantially reducing the incidental mortality of seabirds during longline fishing activities, establishing a CCAMLR Ecosystem Monitoring Program (CEMP) and implementing a range of measures to provide some level of protection for vulnerable marine ecosystems (VMEs). Further, there is no evidence of commercial extinction of any harvested species at the Convention area level since the Convention came into force (CCAMLR, 2008b, 2020b), and severely depleted toothfish and Mackerel icefish stocks have been rebuilt to the level where controlled fishing is now permitted in some areas.^3^

Recent challenges experienced by CCAMLR in reaching agreement on its commitment to implement a network of MPAs, to adopt actions to address the impacts of a climate change and to address Member non-compliance have led to speculations around the effectiveness of the consensus process in the current geopolitical climate (Brooks et al., 2016, 2020; Cavanagh et al., 2021; Chen, 2021; Haward, 2006; Jacquet et al. 2016; Leroy & Morin, 2018; Miller & Murray, 2019; Nilsson et al., 2016; Turner et al., 2008; Wendebourg, 2020).

This paper reviews the level of uptake of Member-driven proposals to test this assertion and then examines examples of proposals that were not resolved within 3 years to analyse the various approaches and processes used by Members to find resolution in such situations.

## Method

A content analysis of all annual meeting reports of the Commission from 1982 to 2021 (CCAMLR, 1983–2021)^4^ was undertaken and the progress tracked of all Member-driven proposals formally submitted prior to a Commission meeting, from proposal submission to outcome.^5^ The analysis excludes proposed notifications to fish^6^ which are generally accepted on the advice of the Scientific Committee. It also excludes proposals arising during meetings of the Commission or its advisory committees unless a paper was subsequently submitted by a Member according to the criteria outlined above as the progression of such initiatives was difficult to track using only the public record.) Scientific Committee consideration of proposals was also reviewed where relevant (SC-CAMLR, 1983–2021). The data generated then provided the basis for a review of the level of uptake of proposals across Members, categories and time taken, and for a deeper analysis of a number of specific examples.

For those proposals where agreement is reached within 2 years, it is assumed that engaged Members participated constructively to amend the proposed text to find mutually acceptable outcomes. Report text suggests that proposals withdrawn within that 2 year period was primarily because of a lack of general support, although no detailed analysis of the reasons was considered in this paper.

Processes used by Members to build agreement where agreement was not possible within 3 years^7^ were then studied across nine case studies representing a range of approaches taken and outcomes:

*Proposals eventually accepted*

1. An example of a stepwise approach presented over several years to reach agreement close to original proposal: The implementation of 100% observer coverage on krill vessels.
2. An example of radical revision to find agreement: Protection of newly exposed marine areas following ice-shelf retreat or collapse.
3. An example of external influence contributing to achieving agreement: The designation of the Ross Sea region Marine Protected Area.

*Proposals withdrawn without resolution*

4. An example of failure to find agreement despite all concerned Parties participating in the process: A trade measure to address illegal, unreported and unregulated fishing activity.
5. An example of failure to find agreement despite full agreement in initial stages: A Climate Change Response Action Work Program.

*Proposals remaining open for negotiation (as of 2021 CCAMLR Meeting)*

6. An example of failure to accept alternate approach: The proposed prohibition of finning of sharks caught as bycatch in the Convention area.^8^
7. An example of non-consensus-building behaviour by two members despite prior consensus commitment to the approach: The designation of a marine protected area in the Weddell Sea domain.
8. An example of refusal to accept the advice of the Scientific Committee by one Member: A multi-member research plan for exploratory fisheries in Statistical Subarea 58.4.

*Veto action by Members incurring a provisional non-compliant status*

9. An example of one Member using their veto to block a non-compliant status and subsequent action against themselves: The proposed sanctioning of Russian vessel *Palmer* for illegal fishing.

For each case study, actions taken by three groups of Members—the proposal’s proponents, other Members who expressed support for the proposal, and those who expressed an alternative or opposing view—were examined. Both actions undertaken to facilitate the process of building collective agreement and those that did not were collated, using a list of questions developed from literature around the use of consensus (MIT-Harvard Public Disputes Program, accessed on 1 February 2021; Innes & Booher, 1999a; Innes & Booher, 1999b; Susskind, 1999; Dressler, 2006; Emerson et al., 2009; Engelberg, 2021). Table 1 lists the questions used for the initial presentation of a proposal.^9^ It was sometimes difficult to glean answers for all questions from the public record. When information could not be gained from other sources^10^ scores were based on the questions that could be answered.

**Table 1:** Questions used to identify collaborative and non-collaborative actions for initial presentation of a proposal

Indicators of consensus-building behaviour		
Question	Yes/No	Para No/s
Did the proponent/s	Yes = 1; No = 0*	
Provide a clear introduction, explain purpose and issue it is addressing?		
Link the proposal to CCAMLR’s business?		
Acknowledge/address alternative views expressed?		
Work to / produce revisions during the meeting?		
Propose/support Intersessional work?		
*Did other participants*		
Actively support the proposal (view recorded in the report)?		
Agree that the proposal was part of CCAMLR’s business (progressed an obligation of the Commission OR delivery of Article II)?		
Provide suggestions, clarifications, information in support of their position or the proposal?		
Propose compromise text OR suggest/participate in mechanisms to facilitate agreement during the meeting (e.g. in-margin meetings)		
Support/propose intersessional work?		
*Did those with alternate views*		
Engage to find a solution/compromise (e.g. provide alternatives, revisions, participate in in-margin groups)?		
Acknowledge the issue as CCAMLR’s business?		
Clearly articulate their concerns?		
Stick to issue and concerns (not introduce other issues)?		
Gain support from other participants (‘I agree with etc.)?		

Indicators of non-consensus-building behaviour		
*Did any Proponent/Supporter*		
Dismiss alternate views without explanation?		
Question the integrity of the position of another Member?		
Refuse to undertake margin discussions or to modify the proposal?		
Justify their position using arguments outside CAMLR business, such as domestic policy pressure?		
Introduce other issues not specific to that under discussion?		
*Did any Member expressing/supporting alternate views*		
Block consent in SC despite scientific evidence, or in SCIC despite unanimous view of all others?		
Question the integrity of the position of another Member?		
Question available science without providing creditable scientific justification (e.g. SC WG discussions, peer-reviewed papers) or Justify their position using non-science-based non-precautionary approach e.g. absence of threat, insufficient scientific information?		
Change their position after previously expressing support/supporting consensus in WG/SC/SCIC?		
Suggest alternatives exist without providing them? raise legal concerns without addressing them?		

The level of support for each case study was also estimated, using an indicative percentage level of support calculated according to Table 2. The estimates are based on references to named Members and groupings used in the Commission reports, such as ‘most members’ or ‘many members. These were considered along with bracketing paragraphs and other context (e.g. paper authors and co-sponsors) that give clues on the views of other engaged members, participant observation knowledge of preceding discussions not reported directly in the final report.^11^

**Table 2:** Allocation for assessing (indicative) levels of support based on terms used in Commission reports

% Support	No. of members	Terms used in commission reports
90	> 20	‘Reference in Report to ‘Most Members’ in combination with references in the report to support from named Members or explicit reference to no more than three opposing Members
80	20–16	‘Most Members’
60	15	‘Many Members’ or ‘The majority of Members’
50	14–11	‘General support’
40	10–9	‘Several Members’
20	5–1	‘Some Members’ (if no Members are specifically named)
< 10	1–3	One, two or three Members, generally named in the report

Proposals were also considered against issue, based partly on the categories adopted by CCAMLR for the allocation of decisions, known as Conservation Measures,^12^ as listed in Table 3 (CCAMLR, 2021).

**Table 3:** Categories used by CCAMLR to classify conservation measures

Category	CCAMLR CM	Inclusions
Compliance	10	Compliance of Members, their vessels and nationals; compliance of Non-Contracting Parties; measures to combat illegal, unreported and unregulated fishing (IUU); compliance evaluation procedure (CCEP)
General fishery matters	21–26	Notifications of intended fishing; gear regulations; data reporting; research and experiments; minimisation of incidental mortality; environmental protection
Fishery regulations	31–33	General measures; fishing seasons, closed areas and fishing prohibitions; bycatch limits
Finfish regulations	40–43	General; toothfish; icefish; grenadiers
Crustacean—krill fishery regulations	51	Krill fishery regulations
Protected areas	91	CCAMLR Ecosystem Monitoring Program (CEMP) sites; Antarctic Specially Managed and Protected Areas (ASPAs, ASMAs); CCAMLR marine protected areas (MPAs)
Inspection	10	System of Inspection (SOI) established in 1989
Climate change	No specific CM category	There is no specific CM category for climate change response actions. One resolution has been adopted. No CMs or response workplan has been adopted as of 2020
Organisational	Procedures	Measures or procedures adopted to facilitate the organisation of meetings
Other	Resolutions, CMs, procedures, Commission actions	Boundary change proposals, best available science, capacity building, conservation strategy, Convention text amendment proposal, cooperation, data confidentiality and use, performance reviews, safety of vessels and observers

## Results

### Overall uptake

**Fig. 1:**
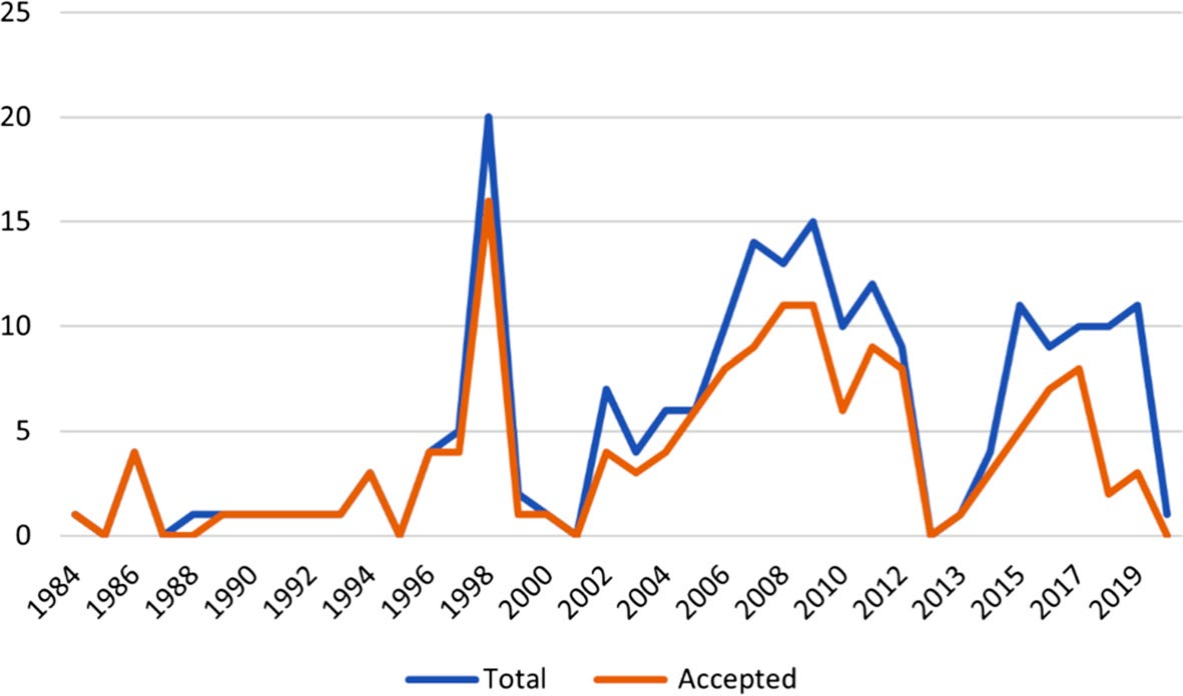
Accepted proposals against total number of proposals presented by year

**Fig. 2:**
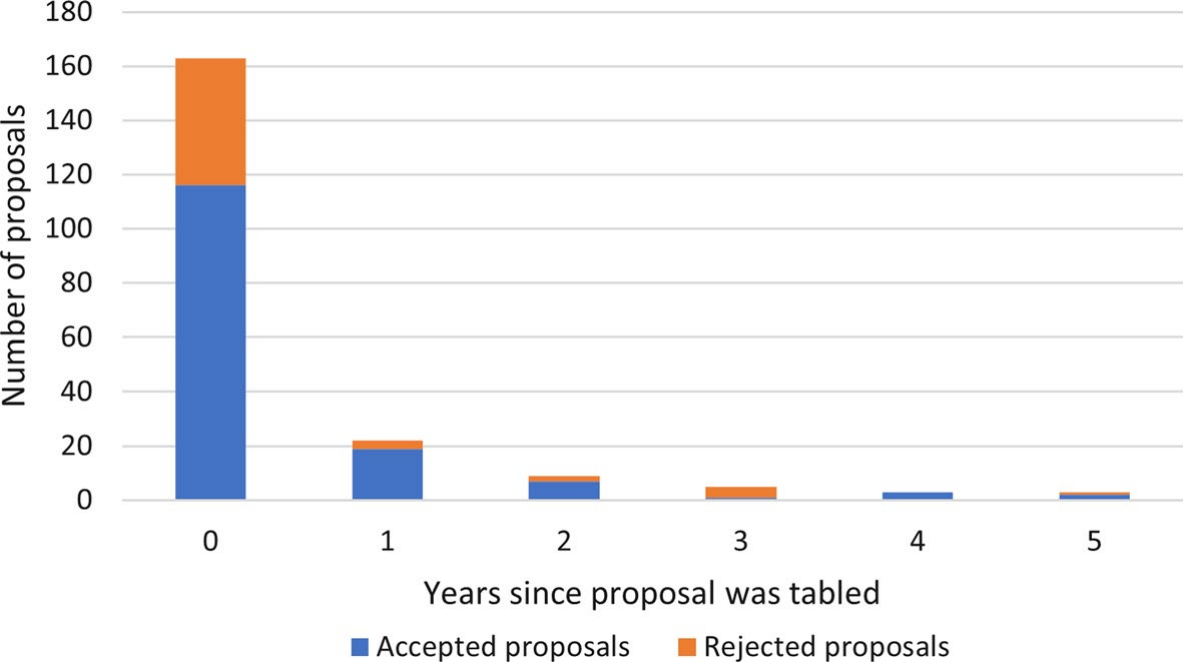
Length of time taken for proposals to be accepted or withdrawn

**Fig. 3:**
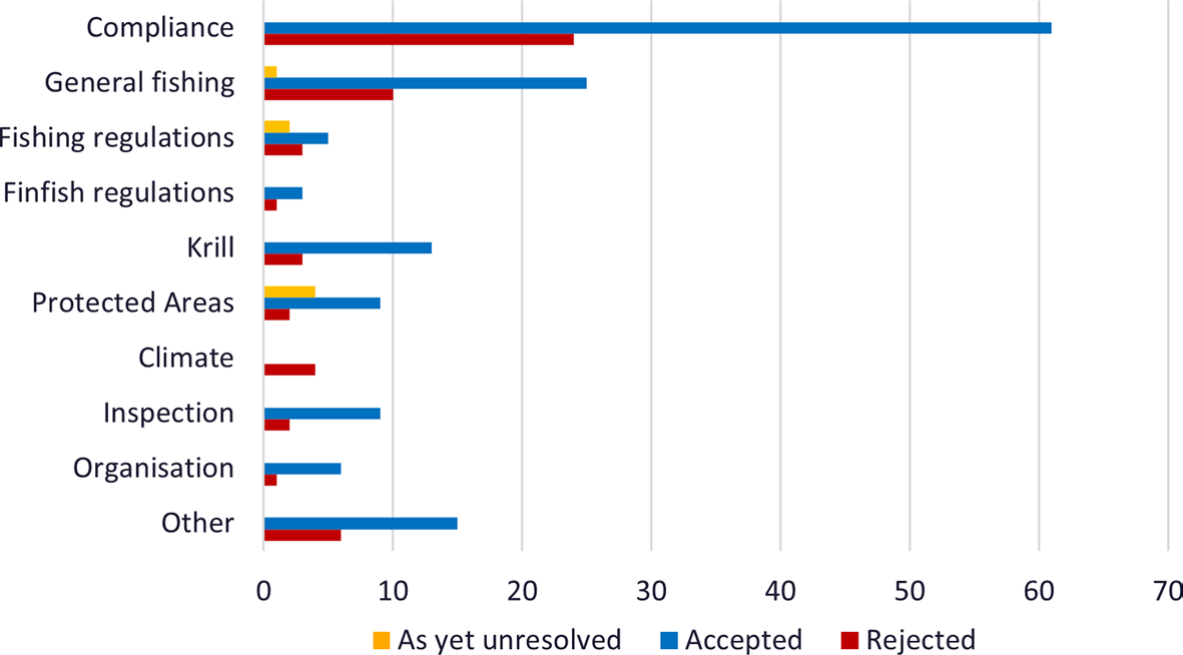
Number of proposals accepted, withdrawn and under discussion according to issue for the period 1982–2020

A review of the outcomes of Member proposals submitted to the Commission between 1982 and 2019 shows that most proposals are taken up after negotiated amendment^13^ and generally within two meetings (Figs. 1 and 2). Equally, decisions to not progress a proposal are generally made quickly, although in more recent years some proposals have remained on the agenda for more than 3 years and at least five have not been resolved but remain on the table after 5 years of discussion^14^ (Fig. 2). Figure 3,^15^ illustrates the number of proposals accepted as a percentage of total proposals submitted per category.no proposals specifically presented to address climate change impacts have been accepted. Of other issues, proposals relating to compliance, general fishing matters and the krill fishery show the highest uptake.

**Fig. 4:**
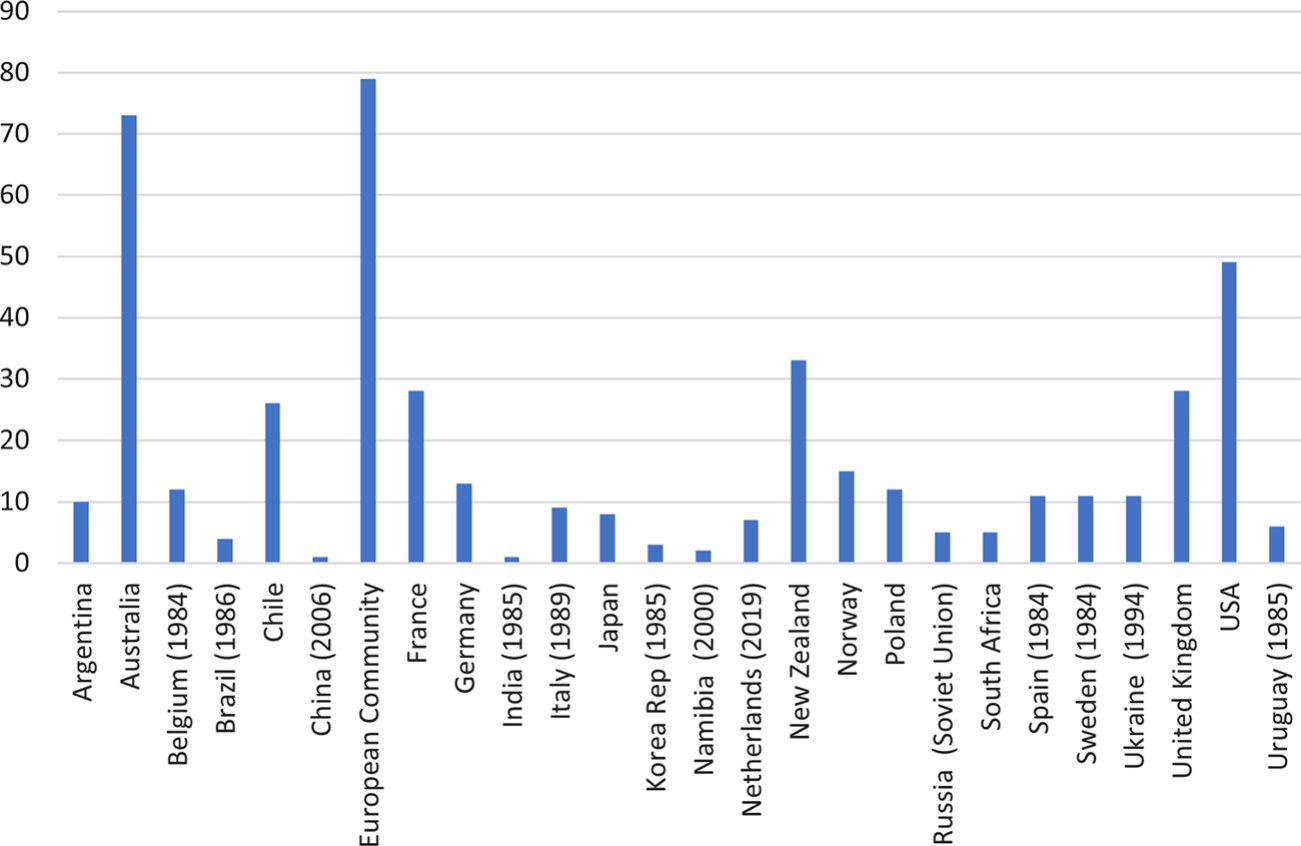
Total proposals per Member per year (includes date of Membership for those who were not founding members)

A small number of Members submit most of the proposals (Fig. 4): four Members have submitted half of the proposals and two-thirds have been submitted by only seven Members.

### Case studies of proposals accepted after several years of discussion

#### Case Study 1: The implementation of 100% observer coverage on krill vessels: an example of a stepwise approach

**Fig. 5:**
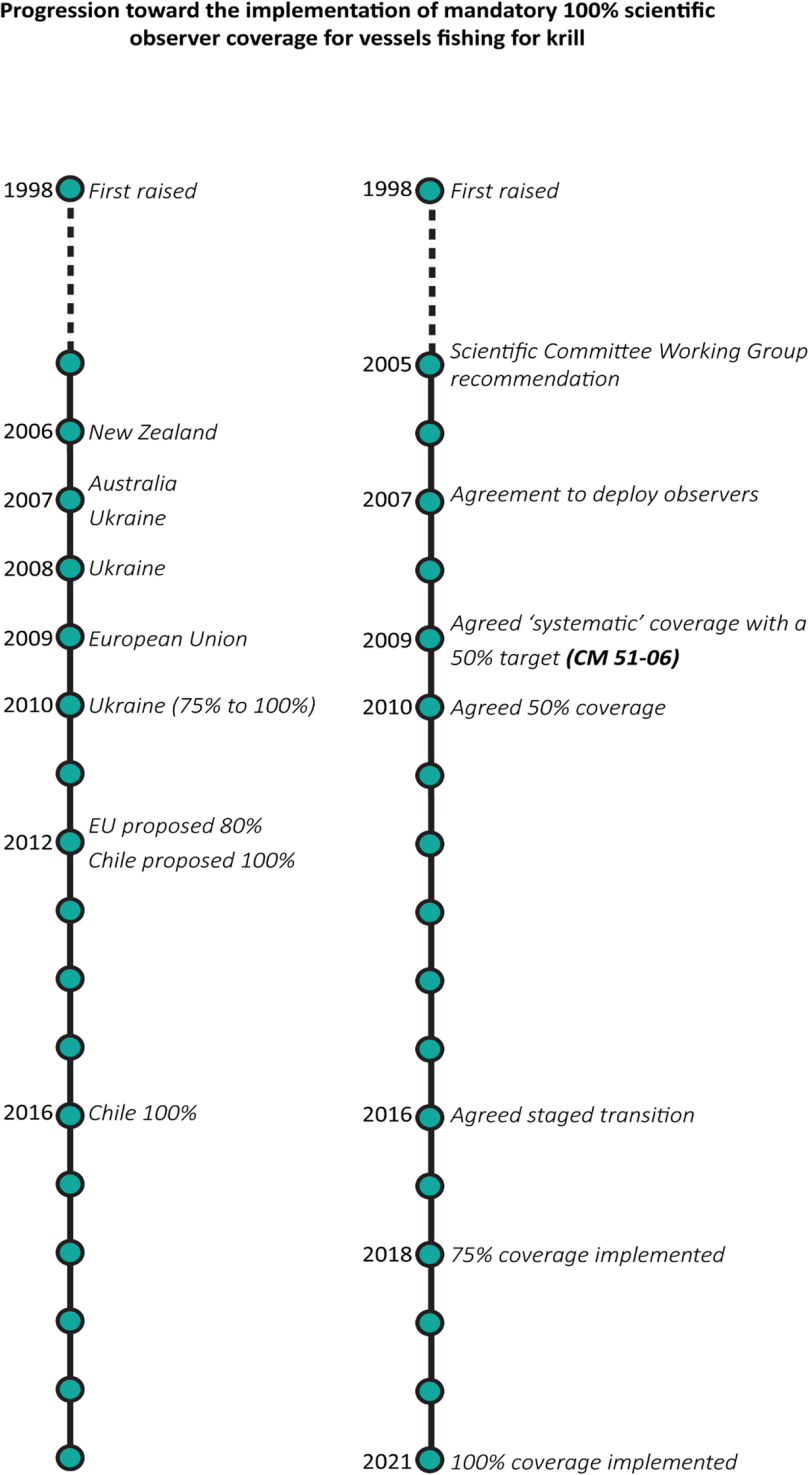
Progression towards implementation of mandatory 100% scientific observer coverage for vessels fishing for Antarctic krill

Mandatory scientific observer coverage for the krill fishery was first raised in 1998 in the context of an agreement for similar coverage for finfish fisheries. However, the then krill fishing nations cited the small size of the fishery as not requiring observers. In 2004, the SC Working Group—Ecosystem Monitoring and Management advised that ‘a consistent level of international observer coverage on krill fishing vessels’ was required to provide the necessary data to adequately assess the fishery (SC-CAMLR, 2004 paras 3.4, 3.29–30), and the SC followed with similar recommendations from 2005 (see e.g. SC-CAMLR, 2005 para 11.6–11.8; SC-CAMLR, 2006 para 2.5–14; SC-CAMLR, 2008, para 6.22–27; SC-CAMLR, 2009, para 6.28–30), noting their preference for 100% coverage.^16^ From 2005, Commission discussions around the proposed 100% scientific observer coverage using CCAMLR-accredited observers for the fishery received considerable support. However, Japan and Korea, and then Japan and China, argued a lack of scientific justification given the low levels of catch and costs to industry, although many vessels hosted national scientific observers (see e.g. CCAMLR, 2005 Annex 5, paras 5.6–7; SC-CAMLR, 2006 para 2.19; CCAMLR, 2006 para 10.4; SC-CAMLR, 2008 para 6.27–31; CCAMLR, 2008a para 4.19–21, 11.7–8). Supporters of the proposal took on a stepwise approach to achieving the desired outcome of 100%. In 2010, the Commission agreed to 50% scientific observer coverage target, but some Members continued to push for 100% coverage and in 2016, the Commission agreed to a phased approach towards 100% coverage by the 2021 season (CCAMLR, 2016 para 8.18) (see Fig. 5).

**Fig. 6:**
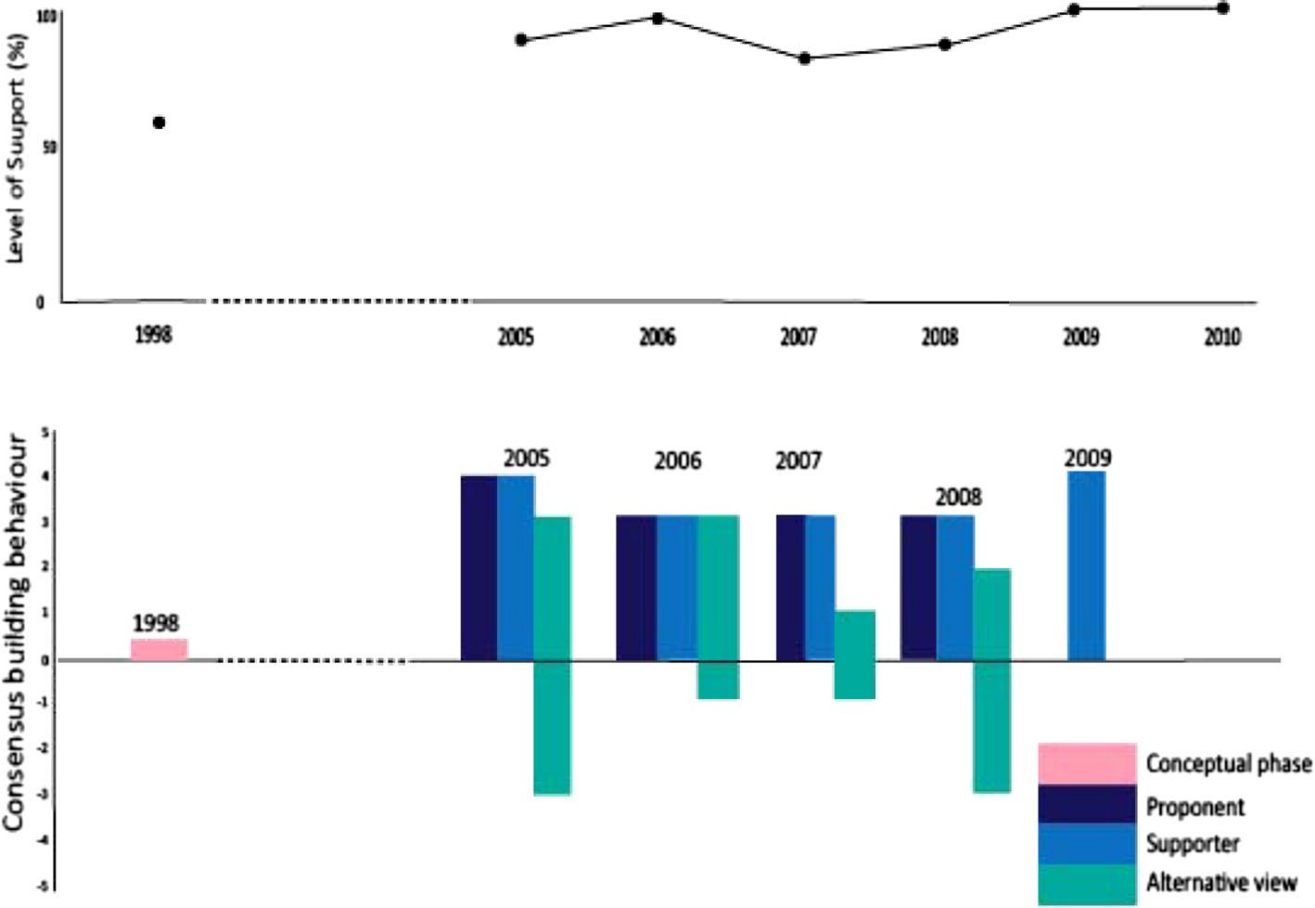
Level of support and consensus-building actions towards the development of mandatory observer coverage for krill fishing vessels

The top row of Fig. 6 shows the level of support for mandatory observer coverage for krill fishing vessels. This indicates very high levels of support from the proposal’s introduction, dropping slightly in 2007, coinciding with consensus agreement to deploy observers without a specified level of coverage or mandated use of CCAMLR-accredited observers, and then steadily building to 2009 when CM 51–06 was agreed, mandating the systematic 50% target coverage rate (CCAMLR, 2009 para 12.59). The strength of consensus-building behaviour (illustrated in the bottom section of the figure) also varied, most likely in line with regrouping as each step was achieved Those opposing the proposal appear to have worked in 2005 and 2006 to find an acceptable way to progress towards mandatory observer coverage and accepted the need for systematic coverage from 2009.

#### Case Study 2: Protection of newly exposed marine areas following ice-shelf retreat or collapse: an example of radical revision to reach agreement

In 2011, the European Union presented a proposal for a marine protected area to protect newly exposed marine habitats as a consequence of the collapse of an ice-shelf, noting that the intent was to be both precautionary and forward thinking, citing the unique opportunity for protection and scientific study of newly exposed habitats undergoing colonisation (SC-CAMLR, 2011 paras 5.67–69; CCAMLR, 2011 para 7.31). Many Members expressed support and noted similar recommendations from both the 2011 CCAMLR MPA Workshop and a recent Antarctic Treaty Meeting of Experts on Climate Change. China and Russia opposed the proposal, citing legal issues, a lack of threat, possible impediment to logistics and the lack of a Research and Monitoring Plan (CCAMLR, 2011). A similar debate ensued in 2012. Despite wide-ranging consultations and extensive revision to a proposal for short-term protection of newly exposed areas for the purpose of scientific research of the unique habitats, the proposal was not agreed and was taken off the table.

Taking into account the concerns expressed, the European Union radically revised the proposal and submitted it in 2015 as a ‘*Time-limited Special Area for Scientific Study (SASS) in newly exposed marine areas following ice-shelf retreat or collapse in Statistical Subareas 48.1, 48.5 and 88.3*’ (CCAMLR, 2015 para 7.1). This version gained immediate support from most Members, but Russia stated it could not support the proposal until further information was available on the exact coordinates and proposed locations for such areas (CCAMLR, 2015 para 7.4). Following revision to further clarify these criteria, the proposal was adopted as CM 24-04 in 2016.

**Fig. 7:**
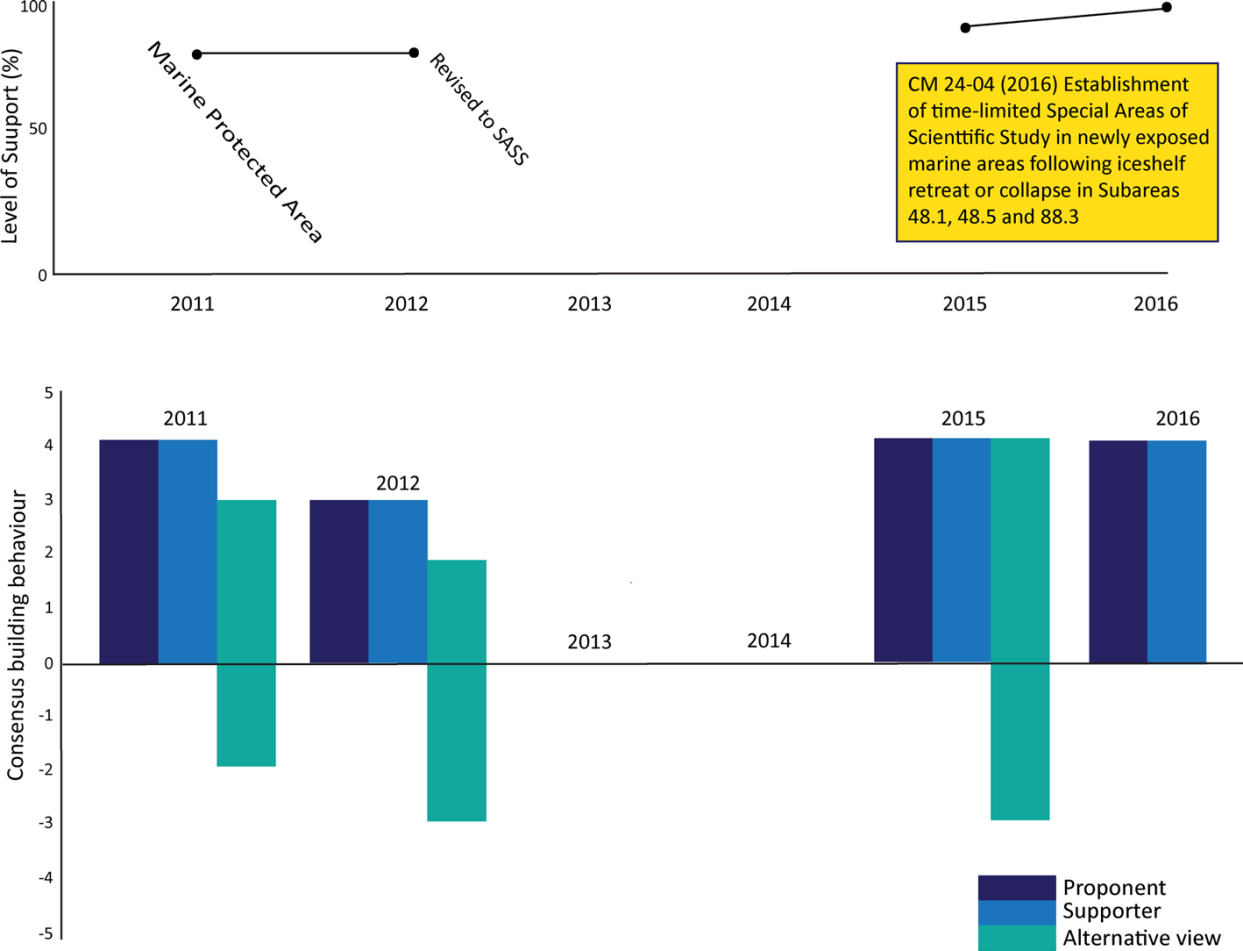
Level of support and consensus-building actions around proposed protection of newly exposed marine areas following ice-shelf retreat or collapse

Figure 7 indicates significant support for the proposal when initially introduced, gaining further momentum when reintroduced in its new form from 2015. The proponents and supporters showed strong consensus-building efforts across most years, and while those initially opposed to the proposal exhibited non-collaborative behaviours across 2011, 2012 and 2015, they also worked hard to find to find agreement in 2015 and 2016.

#### Case Study 3: The adoption of the Ross Sea region MPA: an example of high-level diplomatic influence

After initially presenting separate proposals for the designation of a marine protected area in the Ross Sea region in both 2011 and 2012, United States of America and New Zealand combined their proposals during the 2012 meeting (CCAMLR, 2012 para 7.69). The proposal outlined several objectives, including conserving unique and representative biodiversity and ecological structure and function, promoting research relevant to CCAMLR’s objective, and prohibiting commercial fishing over a large area.

In 2013, the Scientific Committee advised that the proposal had largely incorporated scientific recommendations made at a special intersessional meeting held earlier that year, and there was general although not unanimous support for the scientific elements of the proposal (SC-CAMLR, 2013 paras 5.47–48). Significant compromise revisions were made each year in response to consultation, and both proponents undertook significant high-level diplomatic work, but it was not until the US Secretary of State, John Kerry became involved (U.S. Department of State, 2016) that the proposal adopted in 2016. Brooks et al.,(2021) note that ‘Designating the MPA required the efforts of hundreds of scientists and officials, thousands of conservationists, and millions of global citizens over the course of more than a decade’. The MPA covers 1.55 million km^2^ and includes a large zone off-limits to commercial fishing activity, and two research zones where limited directed fishing for the purposes of fisheries management research are permitted.

**Fig. 8:**
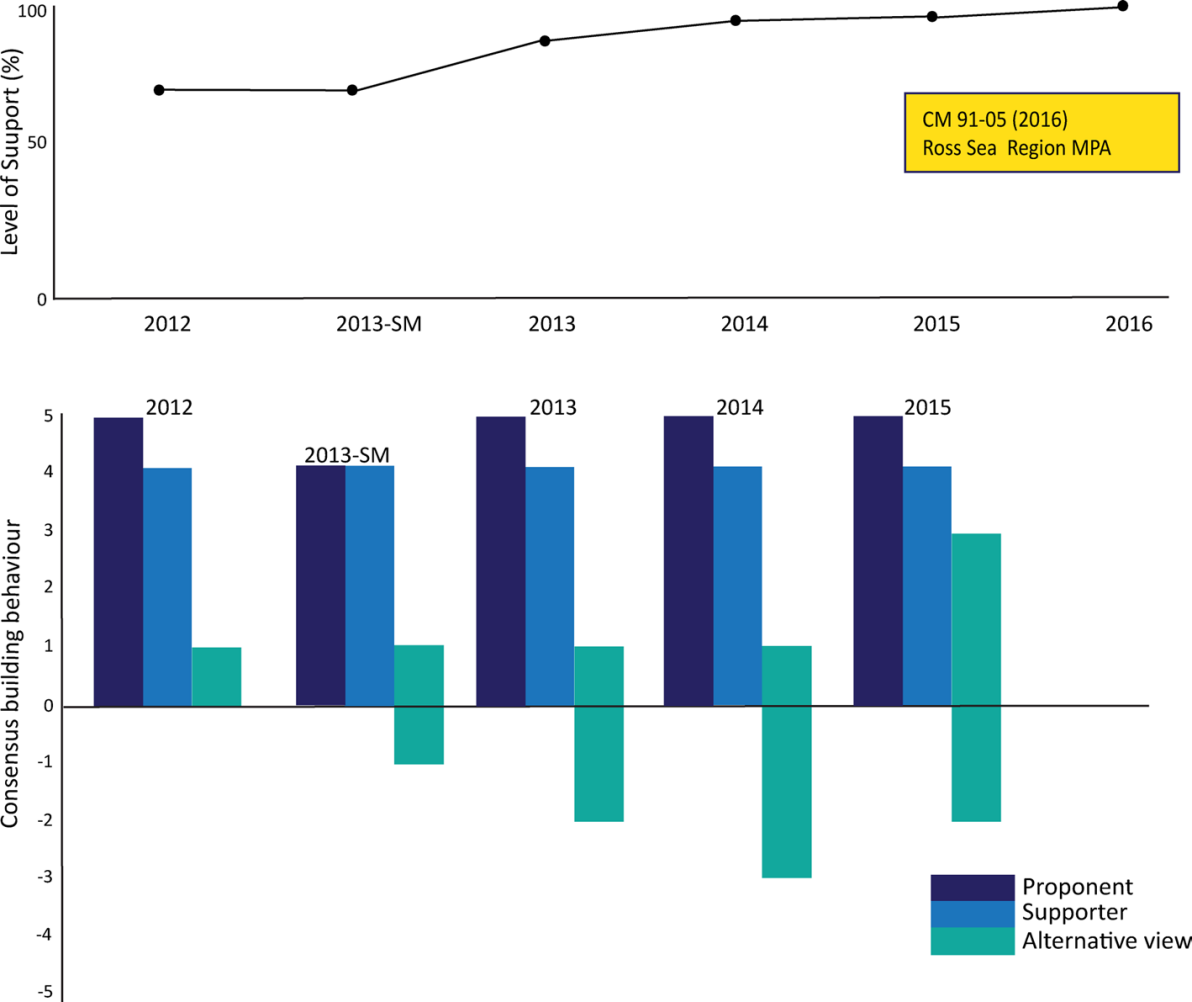
Level of support and consensus-building actions around negotiations for a Ross Sea Region marine protected area

The proposal gained significant support from a strong base established after a Special Meeting held intersessionally in 2013 (CCAMLR, 2013b) and both the proponents and supporters consistently utilised consensus-building approaches to progress the proposal (see Fig. 8). However, those Members with alternative views did not participate proactively in efforts to find agreement until 2015.

### Case studies of proposals withdrawn after several years^17^

#### Case Study 4: A trade measure to address illegal, unreported and unregulated fishing activity: an example of failure to find agreement despite all concerned Parties proactively participating in the process (reflecting differing views on extent of competency of Convention)

The European Union first proposed the use of trade measures to complement existing measures^18^ to combat IUU fishing activity in 2006 (CCAMLR, 2006 Annex 5 para 3.55; Delegation of the European Union, 2006). While the proposal received general support, several Members expressed concerns, including about the competency of CCAMLR to legislate for activities occurring in areas outside the Convention area, and challenges in domestic implementation and in enforcement capacity. Considerable work was done by the proponents in the intersessional period prior to the 2007 meeting, where it gained support from all bar Argentina, who continued to argue that CCAMLR could not impose sanctions on states not party to CCAMLR, as such an approach was contrary to international law (CCAMLR, 2007 paras 10.31, 10.39), a view refuted by many other Members. The proposal was not substantially revised over the next 5 years and continued to be opposed by Argentina (see CCAMLR, 2008a, 2010, 2012). By 2012 Brazil, Namibia, South Africa and Uruguay had joined Argentina in opposing the proposal. In 2013, the European Union submitted a discussion paper to explore how to move forward. Argentina responded that consistent feedback and suggestions for alternative ways to combat illegal fishing had been repeatedly presented (CCAMLR, 2013a para 3.15–16). The European Union made one more attempt to progress the measure in 2014, proposing intersessional discussions. Argentina, with the support of the aforementioned nations, blocked this approach on the basis that there was no possibility of compromise (CCAMLR, 2014 Annex 6 para 233).

**Fig. 9:**
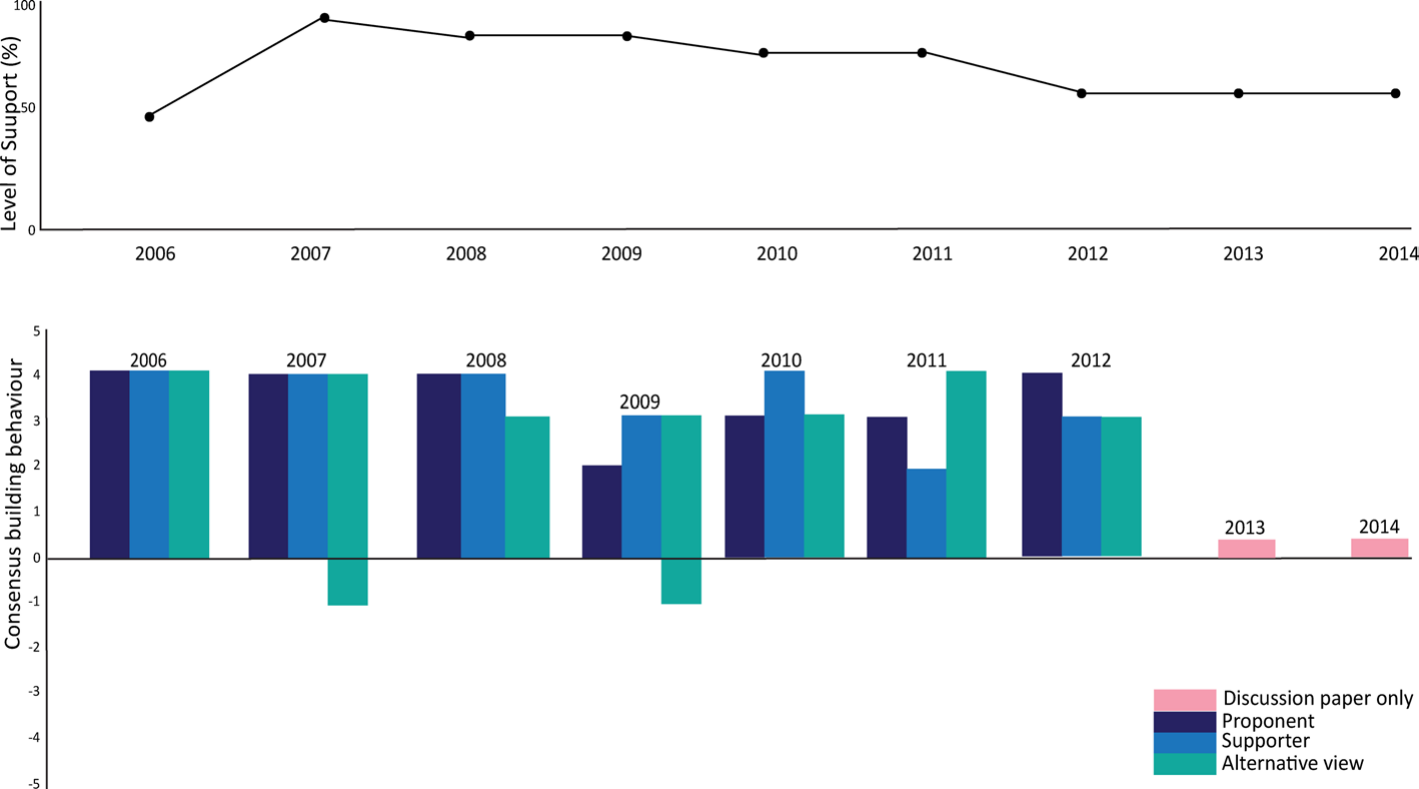
Level of support and consensus-building actions in discussion of a proposed trade measure to address illegal, unreported and unregulated fishing activity

Figure 9 indicates the initial strong support for the proposal declining over time. In the early years of discussion all engaged Members, including those with alternative views, actively worked to find ways forward. However, the proponents extended less effort into building an agreement between 2009 and 2011. Throughout the several years of discussion, those with alternative views continued to display consensus-building approaches while simultaneously building support for their position.

#### Case Study 5: A climate change response action work program: an example of failure to progress despite full agreement in initial stages

From 2003, SC-CAMLR provided information to the Commission on observed and projected impacts of climate change (Goldsworthy & Brennan, 2021). Over several years, the Commission considered, without agreement, a means to integrate this information into its work. In 2015, the Commission endorsed a proposal by Australia and Norway for the establishment of an intersessional correspondence group (ICG) to develop a Climate Change Response Work Plan (CCRWP) (CCAMLR, 2015 para 7.12).

The ICG’s progress report in 2016 was received without adverse comment (CCAMLR, 2016 paras 7.1–8). However, once the draft CCRWP was tabled in 2017, two Members expressed concerns not raised in the ICG discussions, including duplication with work being undertaken in non-CCAMLR forums, and argued that such a work plan would be best located in the Scientific Committee, despite the Scientific Committee’s recommendation that the Commission adopt the CCRWP and its comment that many of the activities identified for the SC and its working groups were already reflected in their 5-year plan (CCAMLR, 2017 paras 7.2–4). The proposal was not presented in 2019, and the 2020 COVID-constrained meeting schedule excluded consideration of proposals outside those necessary to manage active fisheries, although the report reflects several strong Member statements in support of the Commission responding to climate change systematically (CCAMLR, 2020a paras 8.36–41).

**Fig. 10:**
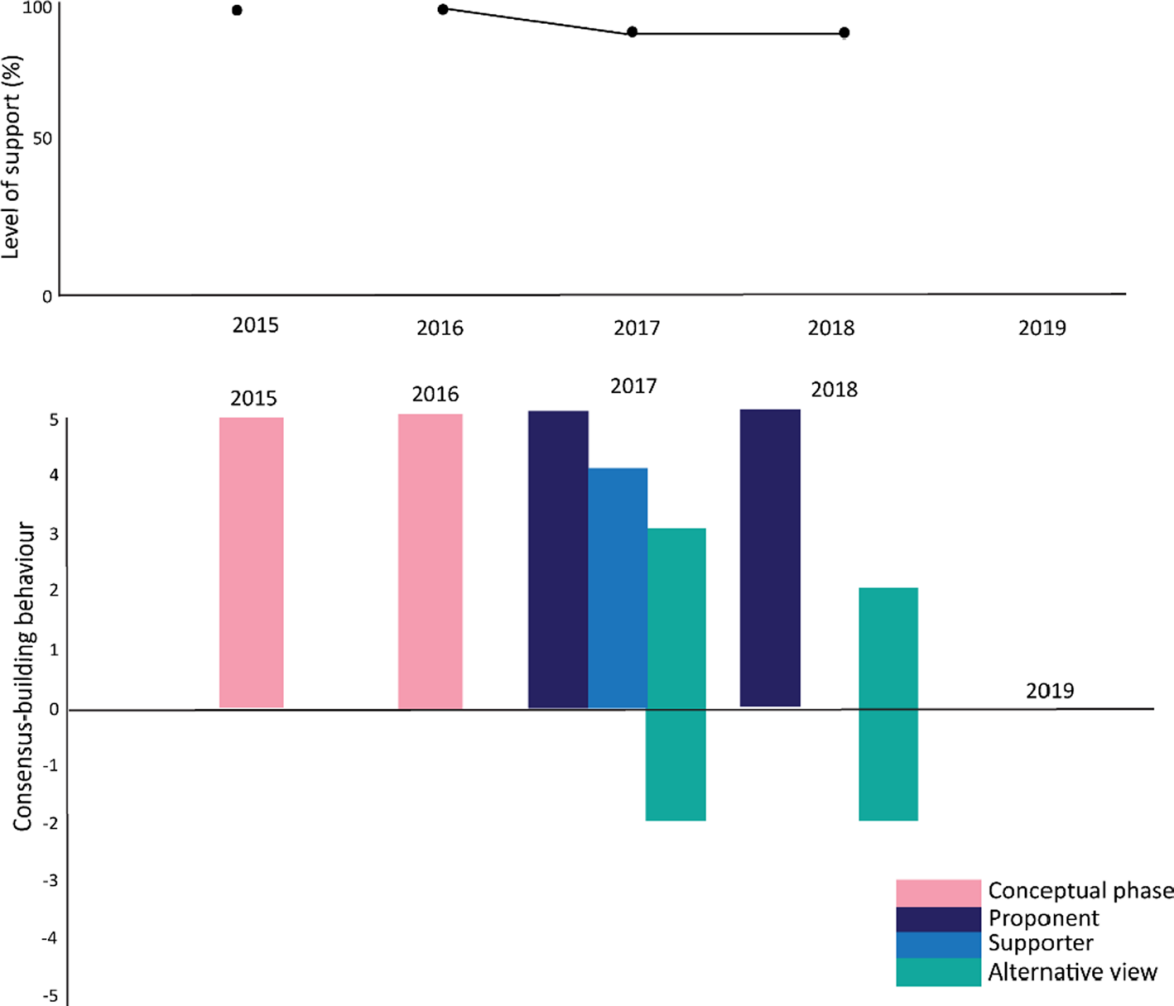
Level of support and consensus-building actions around the adoption of a climate change response work program

Figure 10 shows the high level of support for the proposal—100% when the proposal was at concept stage, dropping to 90% once a specified work program was tabled. The proponents and others utilised consensus-building actions to explore ways forward while the two members who did not accept the proposal applied both consensus-building and delaying actions. The Report text indicated that these members were prepared to accept ongoing scientific research but remained firm in their view that there was no policy role for CCAMLR, even in applying precautionary approaches to ensure the ongoing viability of fishing activity.

### Proposals that remain open^19^

#### Case Study 6: Prohibition of shark finning of sharks caught in the convention area: an example of failure to accept an alternate approach

In 2013, the delegation of the USA proposed amendments to CM 32–18 ‘to require that all sharks incidentally caught in the Convention Area that cannot be released alive be landed with their fins naturally attached’ (CCAMLR, 2013a paras 3.17–18). As directed fishing of sharks was already prohibited,^20^ most Members were very supportive. Japan and China, however, stated that there was no need for such an amendment given bycatch of sharks was very small (CCAMLR, 2013a para 3.22). The proposal was resubmitted in 2014 co-sponsored by Brazil, Chile and the European Union. This year, China stated that bycatch of sharks must be considered legal catch and thus the legal property of those who caught them (CCAMLR, 2014 para 3.66). Japan, expressing the view that the amendments would not contribute to the conservation of sharks in the Convention area, proposed to simply implement a prohibition on shark finning. No consensus was reached on this alternate approach and revisions to the original proposal were presented with varying co-sponsors in 2015, 2016, 2017 and 2019,but without progress towards an agreement. China introduced a new argument in 2016, citing the UNGA resolutions that encourage the full utilisation of dead sharks (CCAMLR, 2016 para 3.27). As of 2019, Japan and China remained the only two Members expressing opposition to the proposed amendments.

**Fig. 11:**
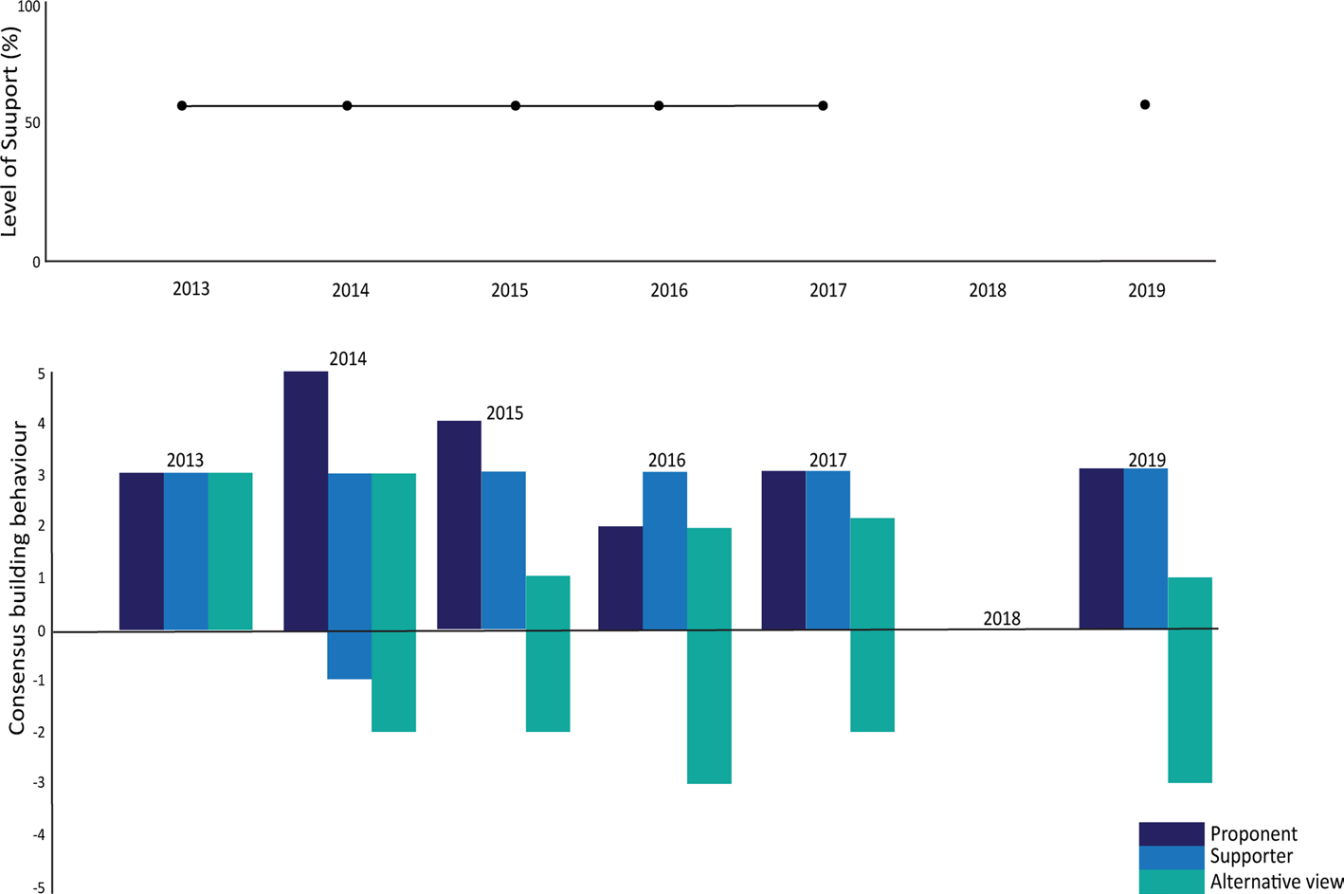
Level of support and consensus-building actions around the proposal to prohibit the finning of sharks caught in the CAMLR Convention area

Figure 11 shows that the level of support for the proposal has remained static throughout the years it has been presented, despite building a significant number of co-sponsors, that consensus-building efforts by all engaged Members has mostly been fair rather than strong, and that opposition had become entrenched by 2016.

#### Case Study 7: The designation of a marine protected area in the Weddell Sea: an example of non-consensus-building behaviour by two members despite prior consensus commitment to the approach

In 2012, Germany took responsibility for the development of a marine protected area in the Weddell Sea as part of the agreed commitment to a representative system of MPAs. In 2013, their progress report was welcomed by many, including Russia, which expressed their willingness to provide, developing a strong scientific basis for the proposal, addressing the various concerns raised through workshops, intersessional discussions and meeting debates. In 2016, the Scientific Committee agreed that the proposal was based on best science available and provided a sound basis for MPA planning in the area (CCAMLR, 2016 para 5.72). The European Union and its member states presented a formal proposal to the Commission in that year. Some Members raised concerns about additional analyses required, whether the proposal did reflect best available science, the need for a fixed duration, and inclusion of fishing vessel-based research (CCAMLR, 2016 paras 5.75–77). Russia introduced new broader concerns, noting that before discussing the specific Weddell Sea proposal, CCAMLR should agree definitions of what constitutes an effective MPA, nature conservation objectives, key ecosystem processes and areas or objects vulnerable to impact by human activities, and determine the criteria needed to evaluate the achievement of each objective (CCAMLR, 2016 para 5.78). By 2019, there was strong support for the adoption of the proposed MPA from all bar China and Russia. It was not discussed in 2020 due to the limited agenda but was submitted and remains on the table.

**Fig. 12:**
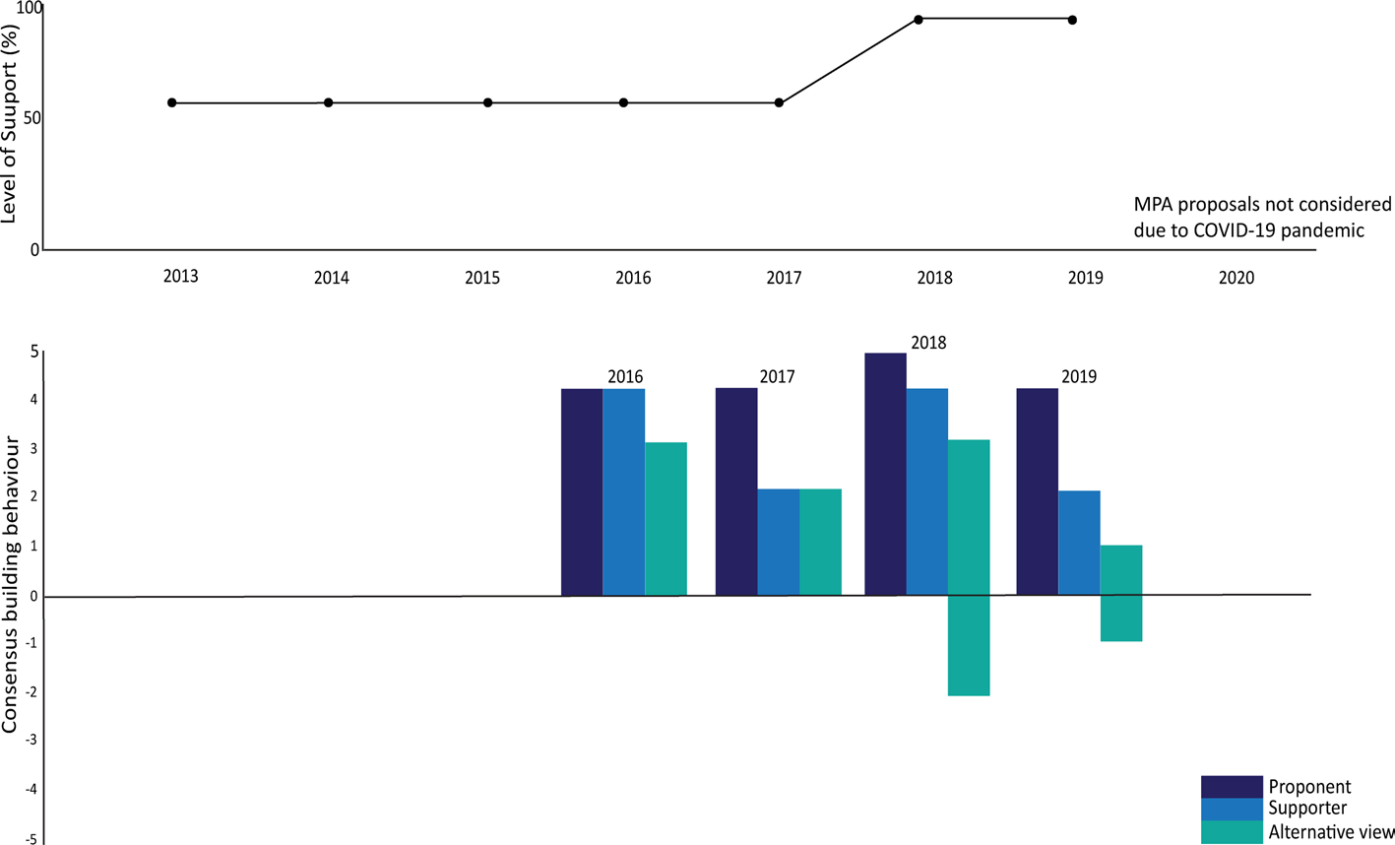
Level of support and consensus-building actions around proposal for a marine protected area in the Weddell Sea

Figure 12 shows momentum has built in support of the proposal alongside strong consensus-building efforts by the proponents and others. Those with alternative views engaged strongly in 2018 but also introduced new conditions that could have been foreseen based on previous discussions.

#### Case Study 8: Proposed extension of a multi-member exploratory fishery and associated research plan: an example of veto used by one member refusing to accept best available science

In 2018, Australia, France, Japan, Korea and Spain submitted results from research undertaken in accordance with their approved research plan as part of their multi-Member exploratory toothfish fishery off East Antarctica. The same group of countries also submitted a proposal for a new 4-year research plan for the same area. However, Russia stated it could not support the new research plan given the different gear types being used by the participating Members (SC-CAMLR, 2018 para 3.137). They maintained this view despite being reminded that the research plan ‘had been extensively reviewed over the last 3 years … and had achieved all research milestones’ (para 3.138), that standardisation methods were ‘used routinely within CCAMLR working groups to control for the potential effects of gear type, vessel, area, depth …’ (SC-CAMLR, 2018 para 3.129), and that many research plans associated with exploratory fisheries used different gear types and vessels (para 3.141). Russia also referenced overfishing in the region in 2007, ignoring the results of the two preliminary stock assessments produced more recently showing that the stock is unlikely to be depleted, even considering historical IUU fishing activity and legal catches. No agreement could be reached in either the SC or the Commission in 2018, 2019 or 2020, despite strong evidence-based support in the Scientific Committee and the only opposition presented in the Commission coming from Russia. Several Members expressed concern about the lack of resolution given the clear scientific advice (see CCAMLR, 2019 paras 5.48–50; CCAMLR, 2020a paras 5.40, 5.42–43), and raised concerns about challenges in providing accurate assessments of the population in the absence of ongoing research and possible increase in illegal fishing activity in the area.

**Fig. 13:**
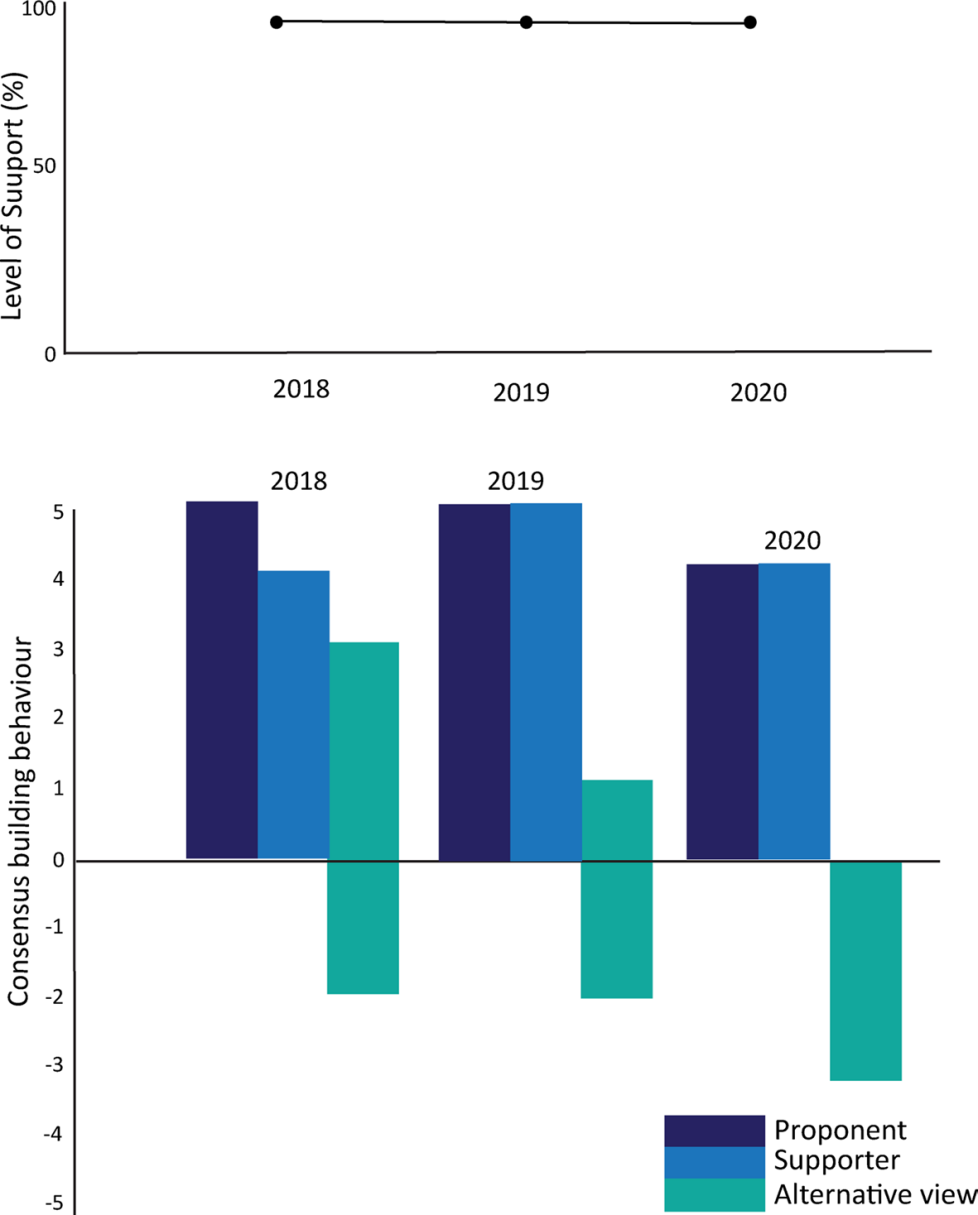
Level of support and consensus-building actions around the proposed extension of a multi-Member exploratory fishery and associated research plan

Figure 13 reflects the discussions in the Commission. Strong support for the proposal, strong efforts to facilitate agreement from many Members and intransigence from Russia.

### Veto action by member incurring a provisional non-compliant status

#### Case Study 9: The proposed sanctioning of Russian vessel Palmer for illegal fishing: an example of a member vetoing action against itself

At CCAMLR (2020a, b), New Zealand reported their observation from a patrol aircraft of the Russian-flagged *Palmer* apparently fishing in an area closed to fishing, some 800 nautical miles from where the vessel was officially reported to be (CCAMLR, 2020a paras 3.5–7). Concluding that the vessel had ‘falsified its VMS data and its entry and exit notifications’, New Zealand proposed that the vessel be included on the CP-IUU Vessel List.^21^ This would effectively prohibit it from fishing in the Convention area. Russia responded that its own investigation placed the vessel elsewhere at that time, questioned the veracity of the images, and proposed that the reported ‘evidence of fishing’ ‘directly contradict[ed] the definition of fishing activity in Article X of the text of the CAMLR System of Inspection’ (CCAMLR, 2020a para 3.12). Both Russia and China also directed the Commission’s attention to their concerns that aerial patrolling was not part of the CCAMLR System of Inspection. Russia refused to release their VMS data for the *Palmer* for further analysis and blocked the inclusion of the *Palmer* on the CP-IUU Vessel List.

They then refused to withdraw notifications for the Palmer to fish in the coming season, citing the absence of evidence to support allegations of the Palmer’s involvement in any non-compliant or illegal activity (CCAMLR, 2020a para 5.26).

## Discussion

In CCAMLR, consensus is taken as the absence of objection, which can sometimes conceal the specific reasons for opposition. A review of CCAMLR annual reports suggests, however, that proposals less likely to succeed are linked to concerns about restrictions on existing or aspirational fishing interests, strongly held national policies, the perceived lack of need for action, or to the potential impact of a CCAMLR measure on activities or discussions occurring outside the Convention area. Proposals most likely to succeed are generally related to fisheries management or compliance. After initially struggling to find agreements uptake of Member proposals was high by the early 1990s and mostly within 1 or 2 years. Cases where there is evidence of a sustained building of support following initial opposition also usually resulted in agreement.

The analysis shows that a small number of Members submit most of the proposals and take the lead in building agreement around these proposals. There appears, however, to be no obligation for others to actively engage in building agreement. While analysis of meeting reports indicates that several Members do constructively participate in the process, others appear to expect solutions to their concerns to be provided by the proponent. Some Members are less actively engaged unless a proposal is specific to their national interests (e.g. Brazil, Japan, India, the Republic of Korea, Namibia, South Africa and Uruguay), and some EU Member states are less active than others (e.g. Italy, Poland, Spain and Sweden).^22^

Japan has consistently maintained a position that measures be adopted based on need. Japan’s slow uptake of full coverage of scientific observers for the krill fishery (Fig. 6) and continued opposition to the US-led proposal to land incidentally caught sharks that cannot be released alive with their shark fins retained (CS-6) are both examples of this view. However, Japan does engage in the process to find practical solutions: it implemented voluntary observer coverage for its krill fishery and eventually accepted a stepwise approach to the implementation of full coverage, and it put forward an alternative, a simple prohibition of finning instead of the substantive amendments proposed by the USA to tackle concerns about removal of shark fins.

Blocking or delaying behaviour by Russia and China is increasingly evident. While this is legally within their rights under the consensus rule, it seems an antithesis to the spirit and intention of the compliance procedure. Further, CCAMLR’s dispute resolution system does not extend to disputes relating to the implementation of Conservation Measures.^23^

China and Russia have both blocked progress on a range of proposals, either alone or in tandem (e.g. non-compliance status against themselves, measures to address climate change impacts, specific marine protected area proposals, the extension of the Pine Glacier Special Area for Scientific Study, the adoption of MPA Reviews and Research and Monitoring Program, and specific fishery notifications).

For China, opposition appears to be most strongly linked to concerns about restrictions to existing fishing activities and to their aspirational fishing interests. For example, China has argued that dead sharks caught incidentally should be ‘regarded as the legal property of the fishermen’ to dispose as they wish (CCAMLR, 2014 paras 3.66–67), also citing UNGA resolutions that encourage the full utilisation of dead sharks (CCAMLR, 2016 para 3.27).

The sudden opposition by China and Russia to an explicit climate response action work program (CS-5) after expressing no concerns during its development, again suggests that concerns about the potential impacts on their fishing expansion aspirations are overriding their commitment to take appropriate measures for the conservation and management of resources in the region and to honour best available scientific advice provided by the SC. The krill fishery may be impacted by seasonal and area closures to protect coastal predators affected by changes in krill distribution and availability.

China’s strong response to what most CCAMLR Members considered a minor administrative oversight came close to resulting in no CCAMLR compliance actions being adopted in 2017. Further, the compromise agreed after reportedly lengthy discussions was not in accordance with the procedures outlined in the relevant CM (CCAMLR, 2017 para 3.30). Several Members expressed their concern about the potential impact on the effective functioning of the organisation if the mutual commitment of all Members to cooperate to seek agreements was not honoured (CCAMLR, 2017 para 3.30–40, 3.43).

Russia appears comfortable in using its ‘vote’ as a bargaining chip to ensure its own vessels can continue to fish despite concerns about non-compliance or insufficient science to support proposed fishery plans. Russia’s continued blocking of the multi-Member exploratory fishery notification (CS-8) also appears to be linked to its own fishing interests in this area.

Russia has a history of blocking the inclusion of its vessels on the CP-IUU List. For example, Russia blocked the inclusion of the *Strela* and the *Zarya* following reports of falsified fishing licenses and/or catch documents in 2003 (CCAMLR, 2003), and the *Volna* from inclusion on the IUU Vessel List in 2006, despite observations of it fishing in a closed area and a widespread support for its listing (Turner et al., 2008). Despite an agreement to reconsider the issue in 2007 following additional investigation by Russia, a license was immediately provided for the *Volna* to fish. No further action was taken, however, as the vessel did not fish in the 2006/2007 season. Members acquiesced to Russia’s refusal to list the *Palmer* on the IUU Vessel list (which would have prohibited it fishing in the CCAMLR area), assumedly to avoid a possible Russian veto on their own fishery notifications.

Chen (2021) suggests that Russia’s position may indicate broader discontent with CCAMLR, citing an interview with Vasily Sokolov, head of the Russian delegation to CCAMLR, who stated that the *Palmer attack ‘*was a calculated attack in response to Russia’s strong opposition [to the marine protected area proposals advocated by Western nations]’ (RIA Novosti, 2020).

Opposition to MPA proposals also appears to be linked to an interpretation of CCAMLR as a regional fisheries body with no role in biodiversity protection (CCAMLR, 2016 para 9.17; Liu, 2020; Chen, 2021). A small number of Members appear to be comfortable to use their option to oppose a proposal, either alone or in conjunction with a small number of others. For example, Argentina will maintain opposition to any proposal that attempts to extend the competency of CCAMLR beyond its own membership or to activities beyond the Convention area they do not consider to be necessary (e.g. CCAMLR, 2008a para 13.75). This position led to its opposition to the proposed trade measure (CS-2). However, Argentina also actively engages in the process to find alternative solutions and prefers to build agreement for their view rather than stand alone.

MPA proposals generally have struggled to appease the strong existing and aspirational fishing interests of both nations (Brooks, 2013). While the Ross Sea region Marine Protected Area was adopted in 2016, it required significant compromise to meet China’s specific concerns (the inclusion of a new zone for exploratory krill fishing, krill fishing within the research toothfish fishing zone, and an expiration date), in conjunction with an extraordinary level of high-level intersessional bilateral negotiation (Liu & Brooks, 2018). These compromises were also sufficient to gain Russia’s agreement. However, neither nation has accepted other MPA proposals on the table despite inclusion of embedded commercial fishing opportunities and research fishing zones in these proposals, and a general acknowledgement that an expiration date is now a default requirement. Both China and Russia have also agreed to the development of a network of MPAs and to the adoption of a CM to guide the development of MPAs (CM91-04).

In retrospect, the question arises whether China and Russia fully understood the intention of the commitment to implement a system of marine protected areas.^24^ China and Russia both appear to view CCAMLR as a fisheries regime, albeit a highly responsive one. While the use of small-scale seasonal and temporal closures is accepted as relevant fisheries management tools, they appear to see no role for large-scale MPAs for biodiversity protection purposes. Russia’s fishing interests were also negatively impacted by the designation of the Ross Sea region MPA.

## Conclusion

The concept of ‘duty to cooperate’ has been encapsulated in the United Nations Convention on the Law of the Sea (UNCLOS) and the United Nations Fish Stocks Agreement (UNFSA),^25^ which both require their Members to cooperate to ensure conservation and management of the high seas (United Nations, 1982, 2001). All CCAMLR Members are signatories to these agreements and are thus bound by this obligation, although while USA has ratified UNFSA, it has not yet ratified UNCLOS.

Consensus decision-making is central to the work of CCAMLR, and to the broader Antarctic Treaty System. While consensus is the absence of objection, it does not mean unanimity; there is strength in dissent and being able to articulate an alternative position. The process of gaining or building consensus can, as shown in the case studies, be fraught. The analysis in this paper indicates that hardening of attitudes and lack of commitment to collaborative actions to find ground for consensus have affected CCAMLR’s ability to address key issues and will likely mean increasing stresses as Members attempt to pursue action on marine protected areas and climate change responses.

While there are obvious signs of blocking and veto this does not mean that cooperation, collaboration and compromise to find alternative solutions is impossible. The analysis in this paper highlights that what has been a strength, encouraging Members to work together, has also become an ‘Achilles’ heel’ and a potential structural weakness. While CCAMLR has a strong record of adopting Member proposals generally within 1 or 2 years, in recent years it has shown a trend away from its principles of cooperation and mutual commitment to develop management measures based on best available science.

The Commission has struggled to adopt proposals that are perceived to potentially impact on national fishing interests and aspirations, and those adopted have required significant amendments to meet the interests of a small number of Members. Further, even when adopted, operational aspects of the implementation of these measures are frequently being questioned. While it is unlikely that CCAMLR would deviate from its consensus approach, bequeathed to it from the Antarctic Treaty, the analysis presented in this paper suggests that a review of its current decision-making processes, norms and practices to identify new ways forward may be necessary. A recommitment to ensuring all parties act on their mutual obligation to cooperate and find solutions might also be required.

## Data Availability

Data are available from the corresponding author, [LG], upon reasonable request. These data will be available in IMAS Data Portal at https://data.imas.utas.edu.au/static/landing.html from March 2022.

## Footnotes

1 See Article 22.5 of the Convention on the Regulation of Antarctic Mineral Resource Activities (CRAMRA), negotiated by the ATCPs to regulate commercial resource exploitation. While never ratified, CRAMRA has similarities to CCAMLR in that it was negotiated by the Antarctic Treaty Consultative Parties to manage commercial resource harvesting.

2 https://www.ccamlr.org/en/organisation/key-challenges-and-achievement.

3 The exploratory fishery for Dissostichus spp. fishery on BANZARE Bank (Division 58.4.3b) was closed in 2009 following intensive IUU fishing activity in the region (McKinlay et al., 2008; CCAMLR 2009 para 11.11).

4 The 2020 and 2021 meetings were held virtually with significantly shortened allocated time and severely constrained agendas. Proposals presented at these meetings were excluded from the data set to avoid potentially skewing the analysis. However, general discussion reflected in the Reports was included where relevant.

5 Outcome includes withdrawal of the proposal, the proposal remaining active, a Resolution, Conservation Measure or operational procedure adopted, or re-presentation of proposal in another form.

6 These are annual national notifications for new and exploratory finfish fisheries made under CM 21-01 and 21-02 and for all krill fisheries under CM 21-03. https://www.ccamlr.org/en/system/files/e-schedule2021-22.pdf.

7 The review found that most proposals were either accepted (in original or revised form) or withdrawn within 2 years.

8 This paper was not submitted to the 2020 and 2021 covid-affected agenda-restricted meetings. However, there is no indication in the public report that the paper has been withdrawn.

9 These were modified slightly for subsequent presentations of the proposal and are available as Supplementary material.

10 The author was also able to draw from knowledge gained from participation at many CCAMLR and SC-CAMLR meetings, Working and Information papers and direct questions to Members to confirm understandings that could not be gleaned from the publicly available Reports.

11 This latter information comes from 20 years of author participation at CCAMLR meetings, including reference to author’s own notes, and personal communication with Dr Dirk Welsford, deputy Science Director of Australian Antarctic Division, participant on the Australian delegation to Commission meetings from 2007-present and current CCAMLR Scientific Committee Chair (pers comm 10/12/2021).

12 CCAMLR has established several categories to group similar regulations together. The schedule of conservation measures can be found at https://www.ccamlr.org/en/conservation-and-management/conservation-measures. As there were no Member-driven initiatives covering crustacean fisheries other than krill fisheries or mollusc fisheries, these categories were excluded from the table.

13 Amended proposals, including partially agreed uptake which did not immediately generate a new proposal. were categorised as accepted. Most proposals are amended prior to final adoption. Acceptance within 3 years was taken as an indication of collaboration. The paper does not consider the reasons behind withdrawal of proposals within the 3 year period.

14 The COVID-19-affected meeting in 2020 led to a very restricted agenda, which may have impacted decisions to submit revisions of unresolved proposals.

15 ‘under discussion’ means that no outcome has been determined for the proposal and it continues to be presented.

16 100% coverage means that each krill vessel would have scientific observer coverage for the entirety of the fishing season.

17 Proposals considered in 2018 but not resubmitted in 2019 or 2020 were considered withdrawn.

18 These included measures to mark licensed fishing vessels and their gear (CM 10–01), licensing and inspection obligations (CM 10–02), port inspections of toothfish catch (CM 10–03), satellite-linked vessel monitoring systems (VMS) to track the location of vessels (CM 10–04), and a Catch Documentation Scheme for toothfish catch (CM 10–05).

19 Proposals considered in 2019 but not resubmitted in 2020 were also considered ‘open’ given the restricted agenda in that year.

20 CM 32–18 was adopted in 2006 and bans directed fishing on shark species in the Convention area other than for scientific research.

21 Contracting Party—Illegal, Unreported and Unregulated Fishing Vessel List.

22 This assessment is based on attribution of interventions to specific Member states, references to Members reporting back from informal discussions, the number of proposals submitted by each Member, and direct observation in meetings.

23 Article XXV (1) of the CAMLR Convention specifies that the dispute settlement procedure is limited to ‘interpretation or application of the convention’.

24 See for example statements made by China and Russia under sub-agenda items ‘Marine protected areas’, ‘Proposals for marine protected areas and special areas’, ‘Review of proposals for new MPAs’ and ‘Progress on MPAs’, ‘Development of research and monitoring plans (RMPs) for CCAMLR MPAs’ in CCAMLR annual reports 2012–2020, including extensive debates around inclusions and Member responsibilities for implementing Research and Monitoring plans and calls for amendment of CM 91–04 (2011) General framework for the establishment of CCAMLR Marine Protected Areas.

25 The full title of UNFSA is The United Nations Agreement for the Implementation of the Provisions of the United Nations Convention on the Law of the Sea of 10 December 1982 relating to the Conservation and Management of Straddling Fish Stocks and Highly Migratory Fish Stock.
